# Longitudinal Analysis of BCAA Levels and Hypometabolism in ApoE4‐Positive Alzheimer’s Patients Over A 48‐Month Period

**DOI:** 10.1002/alz.095737

**Published:** 2025-01-09

**Authors:** Jaipal Virdi, Daniel H. Silverman

**Affiliations:** ^1^ University of California, Los Angeles, Los Angeles, CA USA

## Abstract

**Background:**

Alzheimer’s Disease (AD) is a chronic, incurable neurodegenerative condition characterized by extensive systemic, cellular, and molecular abnormalities. One such aspect recent studies have highlighted is the reduction in plasma branched‐chain amino acid (BCAA) concentrations, identifying them as a potential emerging marker for the disease. Although BCAAs have been implicated in the pathogenesis of AD, their utility in clinical prognosis remains unexplored. This study aims to investigate the role of BCAA levels in AD progression, considering the presence or absence of the APOE4 phenotype over a period of forty‐eight months.

**Method:**

Biomarker and clinical diagnosis data for 31 subjects with varying cognitive statuses were obtained from the Alzheimer’s Disease Neuroimaging Initiative (CN, n = 10; LMCI, n = 21). Cognitive status was assessed using the Hypometabolic Convergence Index (HCI), a tool designed to quantify the magnitude and severity of cerebral hypometabolism typical in probable Alzheimer’s Disease patients. BCAA levels were measured through a nuclear magnetic resonance‐based blood biomarker analysis and expressed as concentrations in plasma.

**Result:**

A significant correlation was observed between the initial HCI and BCAA measurements and HCI values taken forty‐eight months later, indicating cognitive decline (n = 31, r = 0.362, p‐value = 0.0383). Stratification by APOE4 phenotype revealed more nuanced insights: subjects with zero alleles showed no significant correlation (n = 16, r = 0.165, p‐value = 0.0542), while those with one or two alleles demonstrated a stronger, tighter correlation (n = 15, r = 0.564, p‐value = 0.0183).

**Conclusion:**

This study corroborates prior research on the association between diminished plasma BCAA levels and reduced neuronal activity in regions pertinent to AD, as reflected by HCI assessments over a forty‐eight month period. Importantly, our findings reveal a significant correlation between BCAA concentrations and cognitive decline predominantly in individuals carrying one or two APOE4 alleles. This observation highlights the potential influence of the APOE4 allele on the impaired transport of BCAAs to the brain, contributing to cognitive deterioration in these groups. Further research is needed to explore the diagnostic value of BCAA levels in broader AD populations and to understand the mechanistic links in non‐carriers of the APOE4 allele.

